# Precision restoration of complex cervical instability and decompression for neurofibromatosis type I: a case report using patient-specific 3D-printed templates

**DOI:** 10.3389/fsurg.2026.1765063

**Published:** 2026-02-12

**Authors:** Zekai Ma, Weipeng Zheng, Ning Wang, Ruicong Li, Shifeng Wen

**Affiliations:** 1 Department of Orthopedics, The First Clinical Medical College, Guangdong Medical University, Zhanjiang, Guangdong, China; 2 Guangzhou First People's Hospital, Guangzhou Medical University, Guangzhou, Guangdong, China; 3 Department of Orthopedics, Guangzhou First People's Hospital, Guangzhou Medical University, School of Medicine, South China University of Technology, Guangzhou, Guangdong, China

**Keywords:** 3D printing, cervical kyphosis, cervical spondylolisthesis, neurofibromatosis type 1 (NF1), patient-specific templates (PST), pedicle screw placement

## Abstract

Severe cervical kyphosis concurrent with neurofibromatosis type 1 (NF-1) and large intraspinal tumors presents a dual challenge of oncological control and biomechanical reconstruction. We report a rare case of a 14-year-old male with NF-1 secondary to C1-C6 cervical spondylolisthesis, cervical retroversion, retrolisthesis of the C3 vertebral body (less than 1/2 of the vertebral diameter), C2 dislocation, atlantoaxial rotational dislocation, and a large intradural tumor, which caused acute spinal cord compression and neurofunctional deficits. An integrated surgical strategy combining microsurgical tumor resection with 3D-printed corrective fusion was implemented. Intraoperatively, gross total resection (GTR) of the tumor was achieved, followed by precise pedicle screw fixation assisted by patient-specific 3D-printed guides. This comprehensive approach prevented further neurological damage, and the patient achieved satisfactory clinical outcomes postoperatively. NF-1 is an autosomal dominant genetic disorder with an incidence of 1/3,000–1/4,000, characterized by diverse manifestations including skin abnormalities, neurological tumors, and musculoskeletal abnormalities. Approximately 50% of cases arise from *de novo* mutations.

**Case description:**

A 14-year-old Han male student with no prior medical history was admitted for a 1-year history of a large right neck mass (15 × 9 cm) and 2 months of progressive generalized weakness. Physical examination revealed multiple café-au-lait macules (>30 mm) and a large cervical mass, consistent with NF-1. Neurological assessment showed decreased muscle strength in all limbs (grade 3), indicating incomplete spinal cord injury. Imaging (MRI/CT) demonstrated an intradural-intramedullary tumor extending through the intervertebral foramen, causing severe spinal cord compression and multiplanar instability (C1–C6).The patient was diagnosed with NF-1, severe cervical kyphosis, and an intraspinal tumor. He underwent posterior decompression, microsurgical GTR of the tumor (confirmed as neurofibroma via intraoperative frozen section), and occipitocervicothoracic fusion assisted by 3D-printed guides. The integrated surgery successfully restored spinal stability and decompressed the spinal cord.

**Conclusion:**

Severe cervical retroversion combined with large NF-1 tumors is rare and technically demanding. A precision-medicine-based model integrating tumor management and digital orthopedic technology is essential for such high-risk cases. Preoperative planning using 3D digital technology enabled precise screw placement, while microsurgical techniques ensured safe and complete tumor resection. This integrated strategy effectively prevented permanent neurological deficits and resulted in satisfactory postoperative outcomes.

## 1 Introduction

Neurofibromatosis type 1 (NF-1) is a common neurocutaneous syndrome with a global incidence of 1/3,000–1/4,000 live births, with a slightly higher prevalence in children than in adults (1). Mutations in theNF1gene lead to abnormal tumor growth, resulting in diverse clinical manifestations including skin abnormalities, skeletal deformities, visual impairments, and cardiovascular complications (2–4). Musculoskeletal involvement is common, with scoliosis being the most prevalent (affecting up to two-thirds of patients), typically occurring in the thoracic region (5). Comorbid analysis of scoliosis and NF-1 has shown that patients with both conditions are more likely to develop malignant brain tumors, epilepsy, hydrocephalus, pigmentation disorders, hypothyroidism, diabetes with chronic complications, and coagulation disorders (6). Early detection and treatment are therefore critical for this population.

Although recent advances in targeted molecular therapies, such as MEK inhibitors (e.g., selumetinib), have shown efficacy in reducing the volume of inoperable plexiform neurofibromas (7), they are not indicated as a first-line treatment for acute spinal cord compression with rapid neurological deterioration. For patients presenting with progressive myelopathy and severe spinal instability, surgical intervention remains the gold standard to salvage neurological function (8). The comprehensive surgical goals in such complex scenarios are threefold: sufficient spinal cord decompression, maximal tumor resection to reduce recurrence risk, and restoration of spinal alignment and stability (9). However, achieving these goals simultaneously in the presence of severe dystrophic cervical kyphosis presents a formidable challenge to the spinal surgeon.

While cervical kyphosis is less common in NF-1-related scoliosis, fusion and correction techniques for NF-1-associated cervical kyphosis and lumbosacral spondylolisthesis have been widely reported (9–11). However, cervical retroversion deformity combined with spondylolisthesis secondary to NF-1 is extremely rare (12). Surgical management of severe NF-1-related cervical kyphosis remains challenging even for experienced surgeons. The choice of surgical approach [anterior-only [AO], posterior-only [PO], or anteroposterior [AP] combined] is individualized based on age, gender, deformity severity, and stiffness, with the goal of achieving adequate spinal cord decompression and stable spinal reconstruction (13). The management of severe dystrophic cervical kyphosis concurrent with large intradural tumors in NF1 represents an exceptional technical challenge, carrying significant risks of iatrogenic spinal cord injury and vascular compromise. In such complex scenarios, standard surgical approaches are often insufficient. This report illustrates a comprehensive, precision-medicine-based treatment model that integrates accurate diagnosis, rigorous preoperative simulation, microsurgical tumor resection, and 3D-guided biomechanical reconstruction. We detail the risk-benefit reasoning behind this aggressive strategy and demonstrate how this holistic approach achieved the preoperative goals of neurological salvage and long-term stability.

## 2 Conventional treatment

### 2.1 Conservative therapy

Targeted therapy has made breakthroughs in NF-1-related tumor management. MEK inhibitors (e.g., selumetinib) are approved for symptomatic, inoperable plexiform neurofibromas (PNF) in children with NF-1, reducing tumor volume and improving symptoms, thereby providing a new option for high-risk or inoperable PNF (14). Pain management medications are used to alleviate chronic pain associated with neurofibromatosis. For cervical kyphosis in NF-1 patients, Halo-gravity traction can improve coronal/sagittal curvatures and rotational subluxation in patients with NF-1 and congenital scoliosis (15).

### 2.2 Surgical therapy

Traditional surgical approaches for NF-1-related cervical kyphosis include AO, PO, and AP fusion. Sirois and Drennan conducted a retrospective analysis of 15 patients with NF-1-related dystrophic cervical kyphosis who underwent PO fusion, finding that 13 patients had progressive kyphosis angles postoperatively, with a pseudarthrosis rate of 38% (16). Helenius et al. studied 22 patients with NF-1 cervical kyphosis and reported that AP fusion provided better cervical angle correction than PO fusion (17). Tao Lin et al. compared the three approaches and concluded that AP fusion offered more reliable and superior kyphosis correction with no progression, compared to AO or PO fusion (13).

However, traditional open surgeries are limited by difficulties in surgical field exposure, imprecise osteotomy angle control, and high postoperative complication rates, resulting in poor outcomes and moderate functional recovery (18).

### 2.3 Novel therapeutic approach in this case

Preoperative virtual planning combined with 3D-printed surgical guides was used to address the complex skeletal deformities in this patient. This technique enabled precise, personalized surgical design, allowing the surgeon to avoid vital blood vessels and nerves, reduce C-arm fluoroscopy use, shorten operative time, and minimize trauma. Intraoperatively, the 3D-printed guides facilitated accurate screw placement and fusion, ensuring stable spinal reconstruction and adequate decompression (19).

## 3 Case presentation

A 14-year-old Han Chinese male student with no prior medical, transfusion, or family history of significance presented to our hospital with a large right cervical mass (first noticed 1 year prior) and progressive limb weakness for over 2 months.

### 3.1 History

The patient was previously healthy. He reported a 1-year history of a growing right cervical mass and a 2-month history of worsening bilateral limb weakness. Over the past 2 months, he experienced a 5-kg unintentional weight loss.

### 3.2 Physical examination

**Figure 1:**
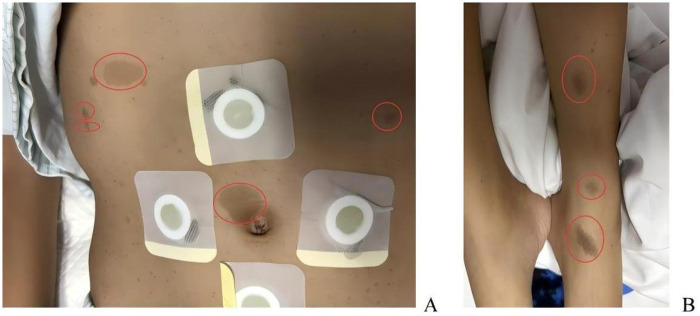
Clinical manifestations of neurofibromatosis type 1. **(A)** Photograph of the trunk showing abdominal café-au-lait macules. **(B)** Photograph of the left lower extremity (lower leg and ankle region). Red circles identify specific lesions that meet or exceed the diagnostic threshold (>15 mm), confirming the criteria described in the text.

Physical examination revealed a 14-year-old adolescent male with secondary sexual characteristics consistent with Tanner stage IV (pubic hair growth and genital development), indicating biological maturity (20). A thorough skin inspection identified a total of nine café-au-lait macules (CALMs). The distribution was explicitly mapped as six lesions on the abdominal wall and three lesions on the left lower extremity (involving the lower leg and ankle).Regarding the size criteria, five large lesions demonstrated maximal diameters exceeding 30 mm, with the largest lesion located at the left ankle exceeding 40 mm. Additionally, three distinct lesions on the abdominal wall exceeded the diagnostic threshold of 15 mm for post-pubertal individuals established by the International Consensus Group on Neurofibromatosis Diagnostic Criteria (21).Furthermore, a large, palpable mass measuring approximately 15 × 9 cm was noted in the right cervical region. Although axillary freckling was absent, the presence of multiple CALMs (>15 mm) combined with the substantial neurofibromatosis mass (plexiform neurofibroma) satisfied the revised clinical diagnostic criteria for Neurofibromatosis Type 1 (21) (Figure 1). Neurological assessment showed hypesthesia below the xiphoid process (positive cotton swab light touch test) and abnormal abdominal reflexes. Motor examination demonstrated normal strength in both upper extremities, intact fine hand coordination, and a negative Hoffman's sign. Both lower extremities exhibited grade 3/5 paralysis, increased muscle tone, and reduced muscle volume. Hyperactive knee reflexes (grade III) and markedly hyperactive ankle reflexes (grade IV) were present, along with positive bilateral Babinski signs. Routine laboratory tests were unremarkable.

### 3.3 Imaging findings

Cranial MRI revealed a small focal area of signal intensity in the left basal ganglia. The lesion appeared isointense on T1-weighted images (T1WI) and hyperintense on T2-weighted images (T2WI) and FLAIR sequences, without enhancement. These findings are consistent with Focal Areas of Signal Intensity (FASIs), characteristic intracranial manifestations of NF1.

Cervical Spine MRI characterized the intraspinal lesion at the C2–C4 level as a solitary mass. The tumor appeared isointense on T1WI and heterogeneously hyperintense on T2WI and fat-suppressed images. Crucially, the tumor exhibited fusiform enlargement along the longitudinal axis of the nerve root, suggesting that the tumor originated from and infiltrated the nerve fascicles. This concentric growth pattern is a hallmark of neurofibromas, distinguishing them from the eccentric growth typical of schwannomas or the dural-based morphology of meningiomas. The tumor demonstrated lateral extension along the course of the right C3 nerve root, passing through the enlarged intervertebral foramen. It caused severe spinal cord compression and myelomalacia. In contrast, extensive soft tissue involvement was observed in the cervical intermuscular spaces and subcutaneous fat layer. These lesions manifested as multiple cystic-like areas with long T1/T2 signals. Unlike the intraspinal tumor, these soft tissue masses showed a diffuse, infiltrative growth pattern consistent with plexiform neurofibromas.

**Figure 2:**
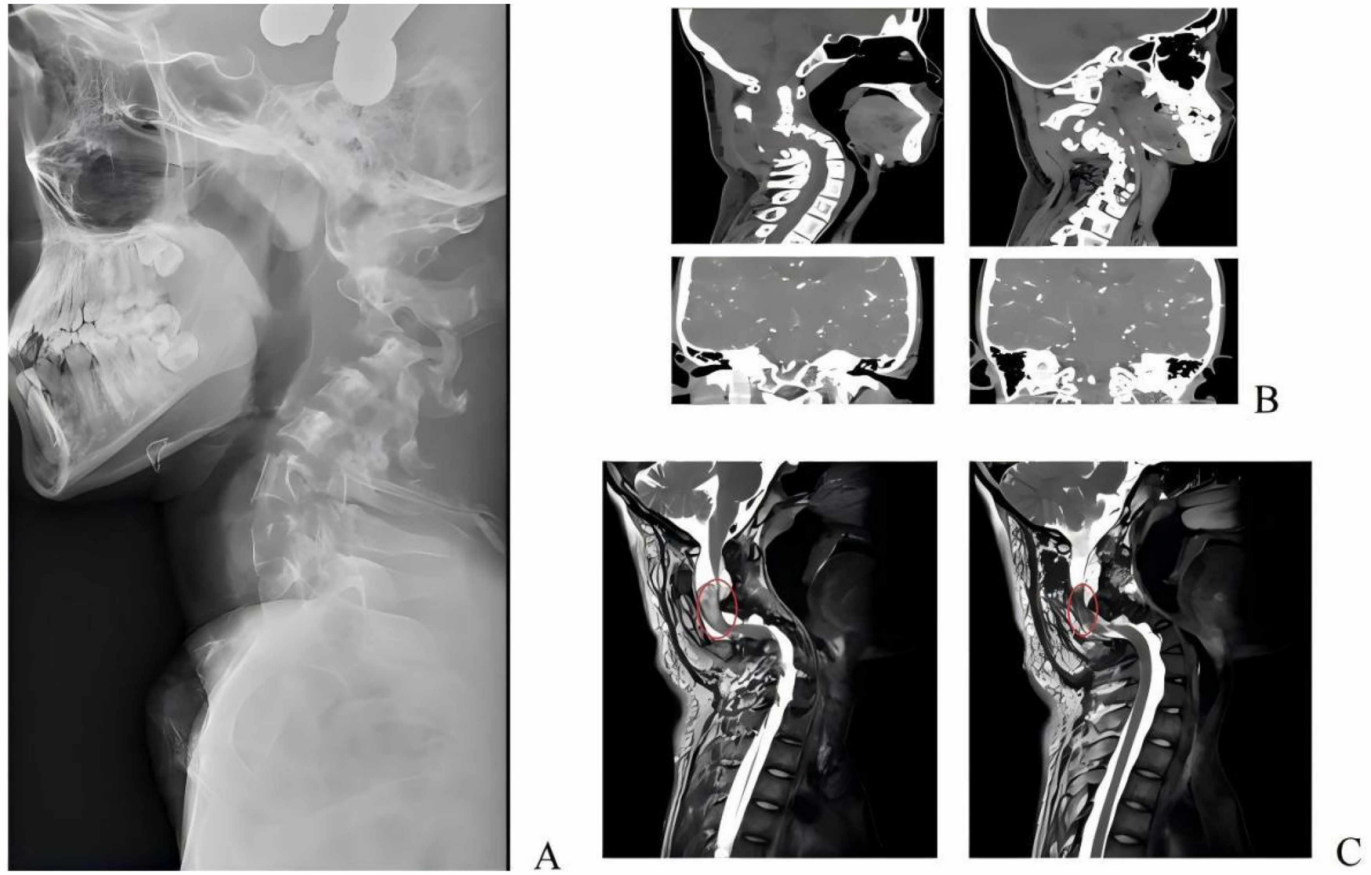
The patient's imaging findings included a cervical lateral radiograph **(A)**, cervical computed tomography (CT; **B**), and cervical magnetic resonance imaging (MRI; **C**), where the tumor location was highlighted with a red circle in the corresponding images.

Plain radiographs and CT scans demonstrated severe dystrophic cervical kyphosis (“reverse arch”) and retrolisthesis of C3 (<50% displacement). The bilateral intervertebral foramina were enlarged, and extensive dystrophic changes (vertebral scalloping, pedicle dysplasia) were noted from C1 to T1 (Figure 2).

### 3.4 Assessment

Comprehensive evaluation identified multi-level cervical instability C1/2,C2/3,C3/4, severe spinal cord compression, and significant distortion of skeletal anatomical landmarks due to NF1-related tumor erosion. These factors classified the patient as a candidate for extremely high-risk, complex cervical spine surgery.

## 4 Preoperative simulation

### 4.1 Preoperative three-dimensional reconstructive structural measurement

**Figure 3:**
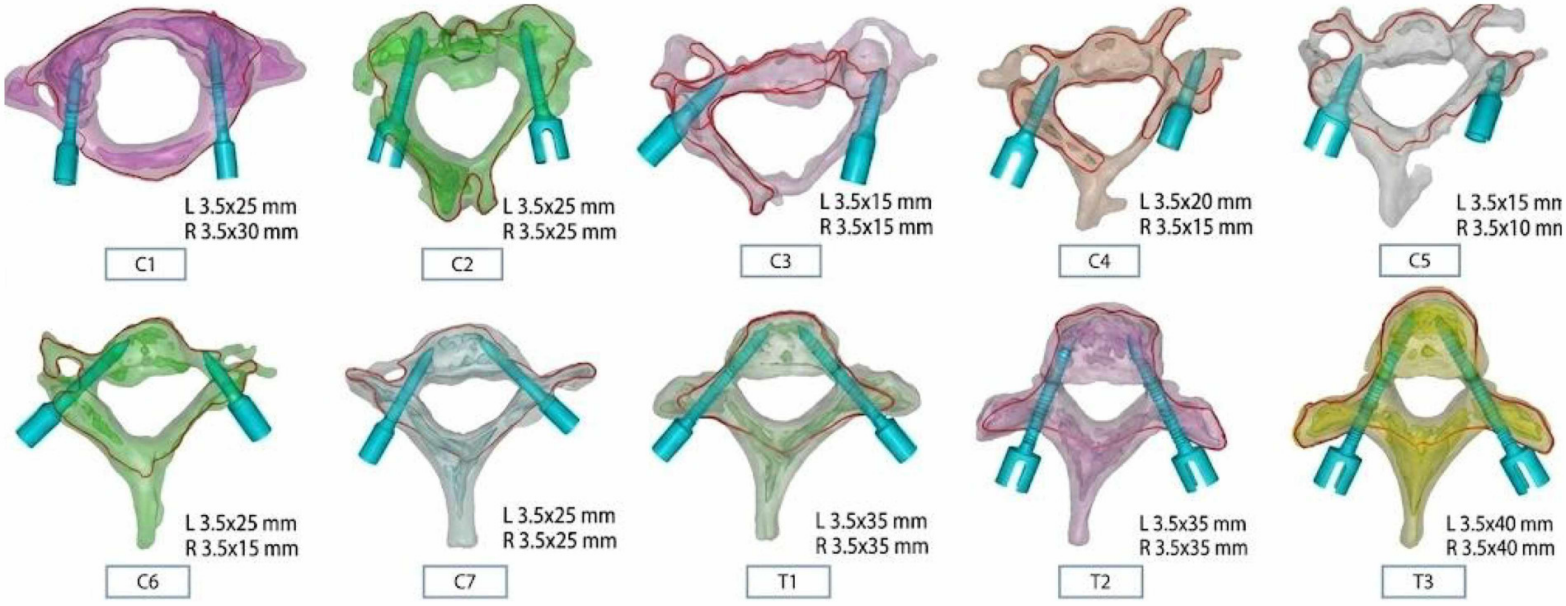
Preoperatively simulated pedicle screw placement angles and sizes for C1-T3 vertebral bodies.

Three-dimensional (3D) reconstruction was performed using preoperative imaging data [x-ray, computed tomography [CT], and magnetic resonance imaging [MRI]] from patients, followed by precise quantification of pedicle length and width for each vertebra from C1 to T3 (Table 1). Digital preoperative simulation was conducted based on the 3D reconstruction data to accurately determine the optimal pedicle screw length and diameter tailored to each vertebra, thereby minimizing intraoperative time consumed by repeated trial-and-error. Leveraging the advantages of preoperative simulation, surgical guides were pre-fabricated and screw specifications were computed. Intraoperatively, the pre-fabricated guides were aligned with the corresponding pedicle screw specifications, ultimately enabling precise surgical execution (Figure 3).

**Table 1:** Preoperative reconstruction of pedicle parameters.

vertebral level	Left pedicle size (mm)	Right pedicle size (mm)
C1	3.5 × 25	3.5 × 30
C2	3.5 × 25	3.5 × 25
C3	3.5 × 15	3.5 × 15
C4	3.5 × 20	3.5 × 15
C5	3.5 × 15	3.5 × 10
C6	3.5 × 25	3.5 × 15
C7	3.5 × 25	3.5 × 25
T1	3.5 × 35	3.5 × 35
T2	3.5 × 35	3.5 × 35
T3	3.5 × 40	3.5 × 40

### 4.2 Preoperative surgical plan design

Option 1: Total laminectomy of C2–C4 and intraspinal tumor resection; occiput–T3 pedicle screw-rod *in situ* fixation (remark: narrowed right pedicles of C3–C5 due to deformity, suggesting C3–C5 lateral mass screw fixation or screw placement avoidance).

Option 2: Total laminectomy of C2–C4 and intraspinal tumor resection; posterior extension reduction of C1–C2 vertebrae; occiput–T3 pedicle screw-rod fixation (remark: narrowed right pedicles of C3–C5 due to deformity, suggesting C3–C5 lateral mass screw fixation or screw placement avoidance).

### 4.3 Preoperative simulation and validation

**Figure 4:**
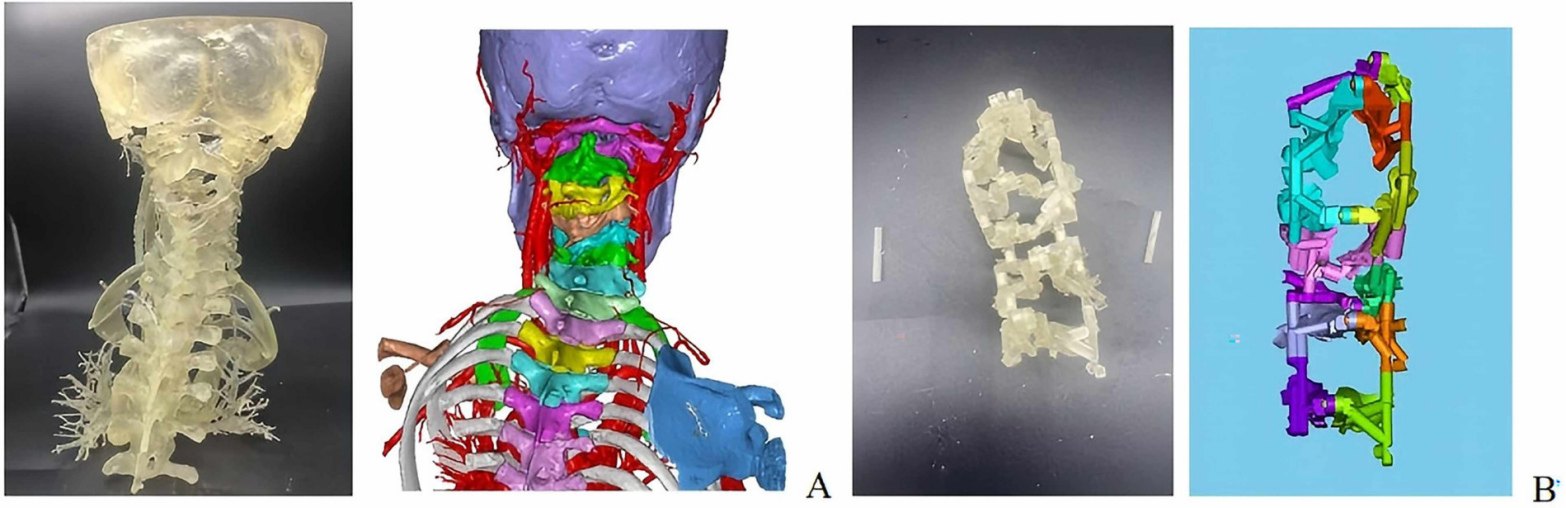
Preoperative preparation of models **(A)** and guides **(B)**, plus 3D printed visualization of the surgical area (including a spinal screw positioning guide device).

To ensure the safety and accuracy of the surgical procedure, a mandatory validation step was incorporated into the preoperative protocol. A 1:1 scale patient-specific anatomical model of the deformed cervical spine was 3D-printed based on the comprehensive 3D reconstruction derived from preoperative x-ray, CT, and MRI data (Figure 4). Prior to sterilization, a preoperative rehearsal (“trial surgery”) was conducted on this physical model. The guides were placed on the model to: (1) verify the perfect congruency between the guides and the bony landmarks; (2) confirm the stability of the guides; and (3) validate the pre-planned screw trajectories. Only after the fit and trajectory were confirmed on the physical model was the workflow approved for intraoperative application.

## 5 Surgical procedure

**Figure 5:**
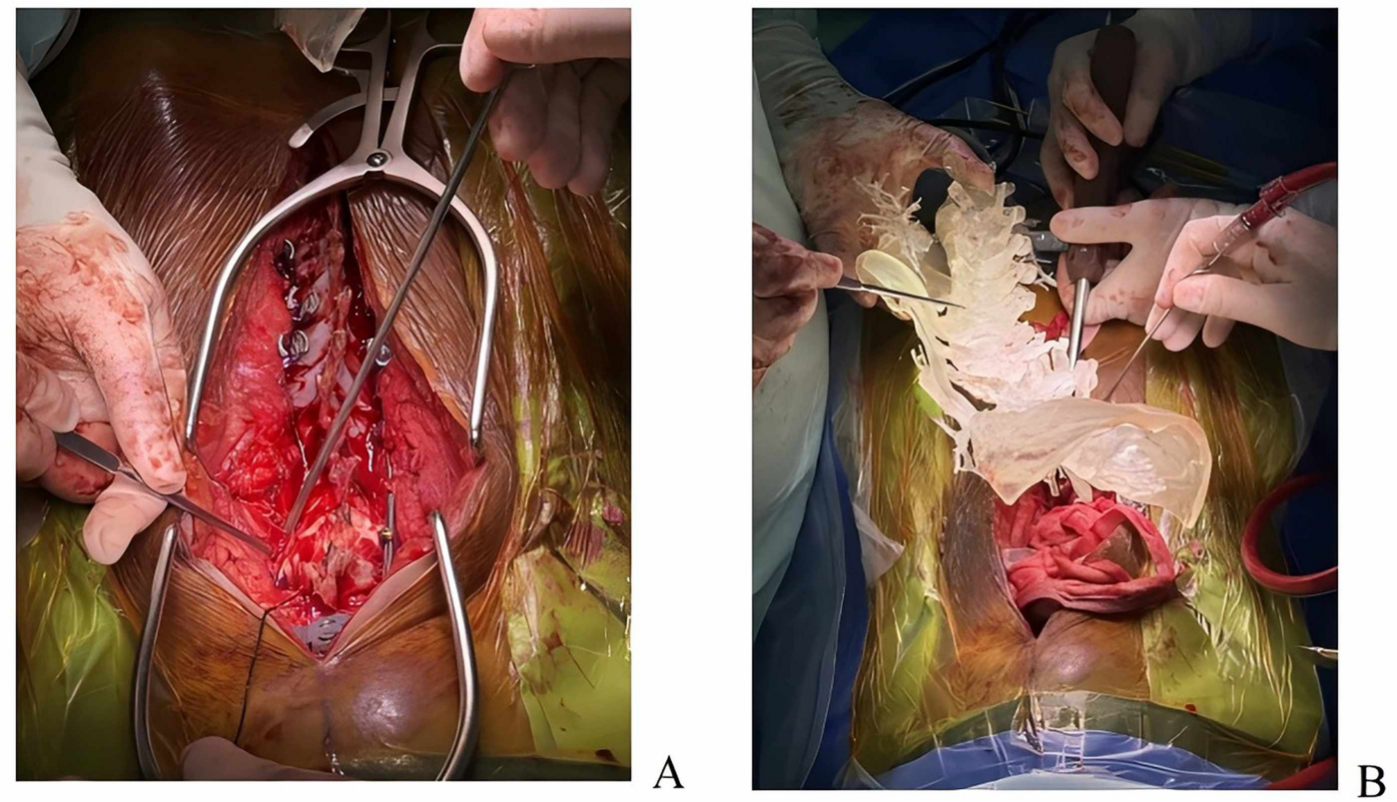
Operative procedure included pedicle screw placement following precise localization via a guide plate **(A)** and intraoperative precise localization using a preoperative 3D-printed patient-specific cervical spine model **(B)**.

**Figure 6:**
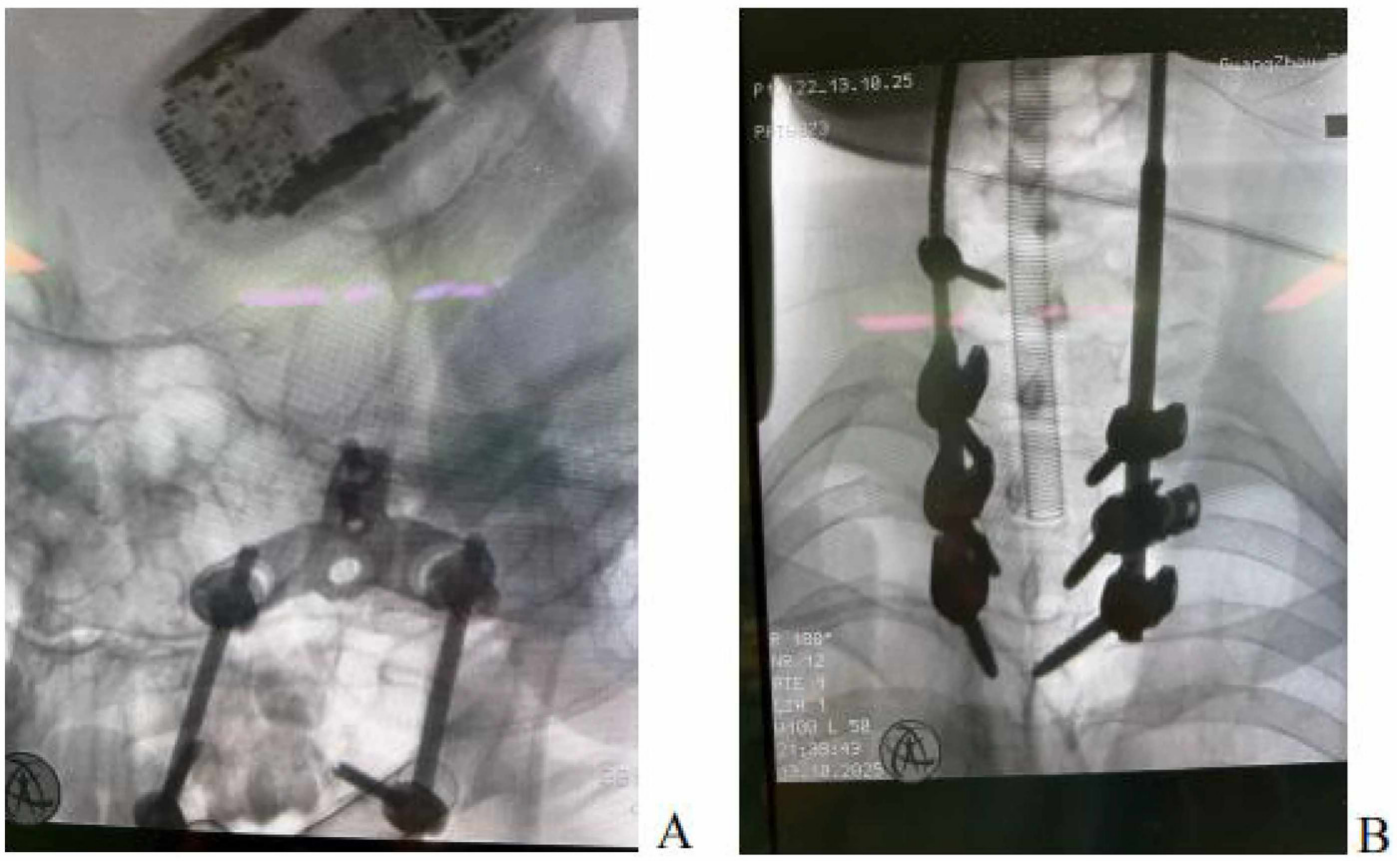
The intraoperative C-arm imaging indicated that the cervical internal fixation nail-rod system was properly positioned **(A)** Upper section of the internal fixation system **(B)** Lower section of the internal fixation system.

**Figure 7:**
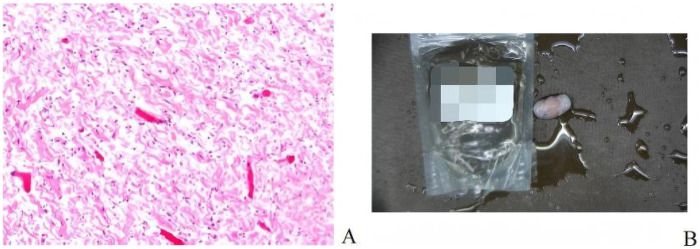
Intraoperative frozen section pathological diagnosis: neurofibroma **(A)** and an encapsulated intramedullary subdural tumor sample (3.5 × 2 × 1 cm) **(B)**.

General anesthesia was administered via endotracheal intubation. Due to severe cervical spinal cord compression, the cervical spine was strictly maintained in a neutral position during intubation, with the assistance of a video laryngoscope to avoid hyperextension. The patient was placed in the prone position, and the head was fixed in a Mayfield head frame, maintaining mild cervical extension to facilitate kyphosis correction. This was performed under neuroelectrophysiological monitoring [somatosensory evoked potentials [SEP]/motor evoked potentials [MEP]] to ensure no decline in neural signals.
After successful anesthesia, the patient was repositioned supine with continuous skull traction, and the surgical field was prepared with routine disinfection and draping.
A midline posterior incision was made from the atlantoaxial region, extending downward to the T4 level, measuring approximately 16 cm. The skin, subcutaneous tissue, and deep fascia were incised, followed by bilateral muscle dissection to expose the posterior arch of the atlas, spinous process of the axis, and articular processes. Intraoperatively, a 3D-printed model was utilized to clarify the patient's anatomical structure (Figure 5). Findings included a loose interspinous ligament of the C3 vertebral body, significant vertebral instability, and a widened C3–C4 spinous process interval. Sterilized 3D-printed guide plates (Figure 4) were secured to the corresponding laminae segmentally. The surgeon confirmed via tactile feedback that the guide plates fit tightly against the bone surface without movement, achieving precise alignment.
Under guidance of the guide plates, a high-speed burr was used to decorticate the cortex at the needle entry points, followed by drilling through the guide holes with a depth-limiting drill bit. Two occipital screws were inserted for fixation. For axis screw placement, the drill was introduced from the dorsal midpoint of the inferior articular process of the axis, passing through the isthmus to the medial aspect of the superior articular process (length: ∼24 mm). C-arm fluoroscopy confirmed optimal screw position (Figure 6). After tapping, a 3.5 mm × 24 mm pedicle screw was inserted with the aid of the 3D-printed guide plate. Additional pedicle screws were placed in the C7 and T2–T4 vertebrae. Pre-bent titanium rods were used to connect the bilateral pedicle screws and occipital screws. Cortical bone was decorticated between the laminae, and a substantial amount of autologous and artificial bone grafts were implanted interlaminarly.
Following total laminectomy and bony decompression, the dura mater was suspended. A midline durotomy was performed under microscopic visualization, revealing a well-circumscribed intradural tumor located subdurally. The tumor demonstrated significant lateral extension along the course of the right C3 nerve root, protruding into the enlarged intervertebral foramen and causing severe compression of the spinal cord medially. Microsurgical gross total resection (GTR) was planned. Using microsurgical techniques, the tumor capsule was carefully dissected from the spinal cord along the arachnoid plane. Intraoperative exploration confirmed that the tumor originated from the C3 nerve root fascicles. To achieve *en bloc* resection and prevent recurrence, the involved C3 nerve root was coagulated and sectioned after confirming it was non-functional due to tumor invasion. The resected specimen was immediately sent for intraoperative frozen section examination. Preliminary histopathological images revealed spindle cell proliferation consistent with a diagnosis of neurofibroma (Figure 7). Primary watertight dural closure was performed using 6-0 Prolene sutures, reinforced with an artificial dural patch and fibrin glue. A subcutaneous drainage tube was placed, and the procedure was uneventful.

The operation was successful, with an estimated blood loss of 800 mL, 200 mL of autologous blood transfusion, and 7,600 mL of fluid infusion. Intraoperative anesthesia was satisfactory, and vital signs remained stable throughout the procedure. The patient was transferred to the central ICU with endotracheal intubation for postoperative resuscitation and intensive care.

## 6 Postoperative three-dimensional reconstruction and postoperative efficacy summary

### 6.1 Measurement of preoperative and postoperative recovery angles

**Figure 8:**
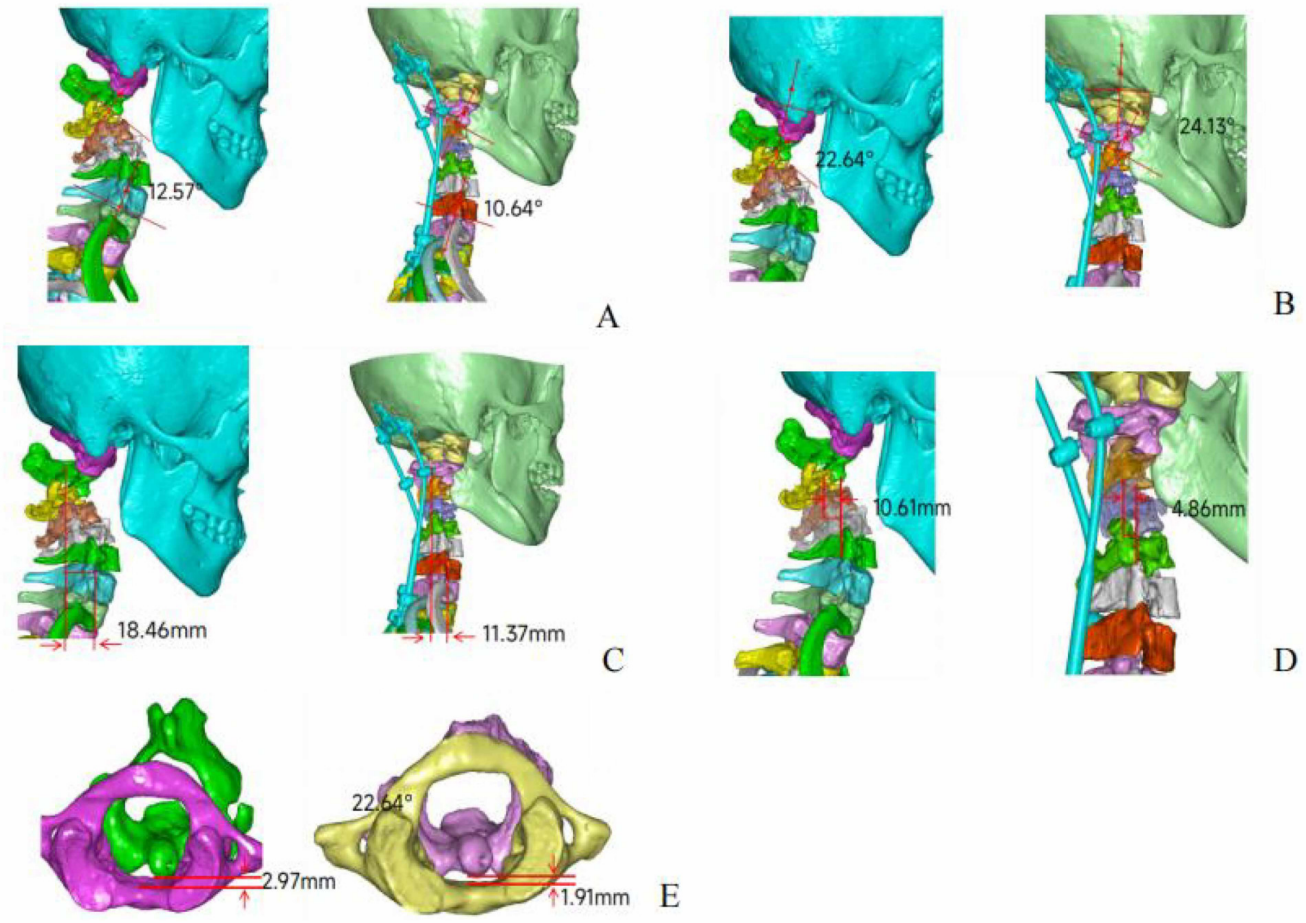
Measurement of various angles before and after cervical spine surgery **(A)** C2-T1 Cobb Angle **(B)** O-C2 Angle **(C)** C2-C7 SVA **(D)** C3 Retrolisthesis **(E)** ADI (C1-C2).

Comparison of five postoperative cervical spine parameters with their preoperative counterparts in patients revealed that all five parameters exhibited improvement relative to the preoperative measurements (Table 2; Figure 8). All postoperative cervical parameters improved compared with the preoperative values. Postoperatively, the patient was safely transferred to the ICU with stable vital signs, and the drainage tube was removed 24 h later. Follow-up x-ray and CT scans revealed restoration of cervical lordosis, almost complete reduction of C3 retrolisthesis, and correction of C1-C2 rotational dislocation. All screws were positioned within the bony channels, without any pedicle wall breaches or vertebral artery injuries.

**Table 2:** Comparison table of measurement parameters before and after spinal surgery.

Parameter	Preoperative	Postoperative	Improvement	Clinical Implication
C2-T1 Cobb Angle(A)	12.57°	10.64°	1.93°	The cervical kyphosis was controlled and the physiological curvature was restored
O-C2 Angle(B)	22.64°	24.13°	1.49°	The upper cervical vertebrae maintained the line of sight level
C2-C7 SVA(C)	18.46 mm	11.37 mm	7.09 mm	The balance of sagittal plane was significantly improved, and the tension of the posterior neck muscles was reduced
C3 Retrolisthesis (D)	10.61 mm	4.86 mm	5.75 mm	The rate of slip reduction was over 50%, and the volume of spinal canal was significantly enlarged
ADI (C1-C2)(E)	2.97 mm	1.91 mm	1.06 mm	The atlantoaxial joint was restored to its normal anatomical position (<3 mm), eliminating the potential risk of high spinal cord compression

## 7 Discussion

Cervical kyphosis associated with neurofibromatosis type 1 (NF1) differs significantly in pathological mechanism from tuberculous or traumatic kyphosis. The “Modulation Theory” proposed by Durrani et al. emphasizes that dystrophic bony changes specific to NF1 not only weaken the biomechanical resistance of vertebral bodies against deformity but also the kyphotic deformity itself exacerbates axial stress on the anterior column, inhibiting anterior vertebral growth and forming a “deformity progression-further bony dysplasia” malignant positive feedback loop (22). The severe posterior dislocation of the C3 vertebra in this patient represents an extreme pathological manifestation of this theory. In such a biomechanical environment, simple posterior decompression without robust three-column fixation is highly prone to accelerating iatrogenic kyphosis due to disruption of the posterior column tension band structure. Therefore, we adopted an occipitocervicothoracic long-segment fixation strategy. Both biomechanical and clinical studies have demonstrated that extending the fixation segment across the cervicothoracic junction (CTJ) to the T2 or T3 vertebra significantly reduces the risk of distal junctional kyphosis (DJK) (23).

Severe cervical kyphosis combined with NF1 and cervical spondylolisthesis is among the most challenging procedures in spinal surgery. Traditional pedicle screw insertion in conventional surgery typically relies on a high-risk free-hand technique, which demands exceptional technical proficiency from the surgeon. Early studies reported a 1.5%–58% incidence of pedicle wall perforation (24–26). With the increasing maturity and precision of 3D printing technology, 3D-printed guides and models are increasingly used as auxiliary tools in routine surgeries (27). However, in complex deformities with severe anatomical disorganization like NF1, they are a critical technology for ensuring surgical safety (1). Accuracy in Screw Placement: The patient exhibited severe right pedicle hypoplasia at C3–C5. In this context, free-hand insertion poses an unacceptable risk of cortical perforation and injury to the vertebral artery or spinal cord. Our patient-specific guides utilized the unique anatomical surface features of the lamina and spinous process for precise registration. Crucially, the reliability of this registration was validated beforehand through preoperative simulation on the 1:1 physical model, ensuring that the intraoperative screw insertion strictly followed the optimal axial trajectory.(2)Oncological Safety and Radiation Reduction: Beyond biomechanical precision, minimizing intraoperative fluoroscopy is of paramount oncological importance. NF1 is a RASopathy belonging to the Cancer Predisposition Syndromes (CPS), characterized by genomic instability and an increased baseline risk of malignancy (21). These patients exhibit a heightened sensitivity to the cumulative DNA-damaging effects of ionizing radiation, which may act as a “second hit” to trigger the malignant transformation of neurofibromas into MPNST (28). By significantly reducing the frequency of C-arm fluoroscopy compared to conventional techniques (29), this guide-assisted workflow serves as a proactive preventive medicine strategy to mitigate long-term oncological risks (3). Improved surgical efficiency: Although preoperative planning and guide fabrication are time-consuming, the elimination of repeated fluoroscopic localization and needle entry point adjustment during surgery essentially shortened the total duration of anesthesia and surgery—this is particularly clinically significant for NF1 children, who often have multiple systemic comorbidities and poor anesthetic tolerance.

The management of NF1-associated complex spinal deformities requires a delicate balance between oncological control and biomechanical restoration. While MEK inhibitors (e.g., selumetinib) have revolutionized the treatment of inoperable plexiform neurofibromas (7), their role in acute spinal cord compression remains limited. According to the 2023 international consensus guidelines for NF1 spinal deformities, early and aggressive surgical intervention is the essential treatment principle for dystrophic deformities presenting with progressive neurological deficits (8). In this case, the patient's rapid deterioration (Muscle Strength Grade 3) necessitated immediate mechanical decompression, a goal that pharmacological cytoreduction—often requiring months to show efficacy—could not achieve in time.

Furthermore, the surgical strategy must address the high risk of recurrence and progression inherent to dystrophic NF1. Recent systematic reviews indicate that for severe cervical kyphosis, robust instrumentation and fusion are critical to preventing post-laminectomy instability (9). By employing patient-specific 3D-printed guides, we achieved safe pedicle screw insertion in dysplastic vertebrae, ensuring immediate stability.

Finally, our treatment protocol extends to long-term surveillance. Given that 8%–13% of NF1 patients may develop malignant peripheral nerve sheath tumors (MPNST) during young adulthood, lifelong monitoring is mandatory (30). We have established a multidisciplinary follow-up protocol involving serial MRI every 6 months to detect any suspicious nodular growth or pain patterns indicative of malignant transformation, ensuring timely intervention if necessary.

Despite the satisfactory short-term outcomes of this surgery, this study has several limitations. First, as a single-center case report, its level of evidence is relatively low. Second, the fabrication of 3D guides involves additional medical costs and a 3–5 day waiting period, making it unsuitable for emergency surgery. Furthermore, the accuracy of screw placement using guides is highly dependent on the quality of intraoperative soft tissue dissection—if incomplete dissection leads to soft tissue entrapment and poor apposition between the guide and the bone surface, severe guidance deviation may occur. Therefore, surgeons must retain profound anatomical expertise and free-hand screw insertion experience and cannot fully rely on digital tools.

## 8 Conclusion

In conclusion, the management of severe dystrophic cervical kyphosis combined with intraspinal tumors in NF1 represents an exceptional technical challenge that demands a systematic, integrated treatment strategy. Although the distorted anatomy posed significant surgical risks, the progressive myelopathy driven by this dual pathology constituted an absolute indication for intervention. Our strategy navigated this high-stakes scenario by strictly prioritizing thorough decompression via microsurgical gross total resection (GTR) to halt further neurological deterioration, while simultaneously mitigating the critical risks of iatrogenic injury and implant failure through rigorous preoperative validation and 3D-guided instrumentation. The successful achievement of robust biomechanical fixation and the effective elimination of tumor compression validate this precision-medicine model, offering a reproducible reference for managing similar complex spinal pathologies.

## Data Availability

The original contributions presented in the study are included in the article/Supplementary Material, further inquiries can be directed to the corresponding author.
